# Association between Serum Biomarkers and Peripheral Neuropathy in Microscopic Polyangiitis

**DOI:** 10.3390/ijms232113374

**Published:** 2022-11-02

**Authors:** Yuichi Masuda, Shogo Matsuda, Takuya Kotani, Daisuke Nishioka, Shin Ota, Takafumi Hosokawa, Shimon Ishida, Tohru Takeuchi

**Affiliations:** 1 Division of Neurology, Department of Internal Medicine (IV), Osaka Medical and Pharmaceutical University, Osaka 569-1094, Japan; 2 Division of Rheumatology, Department of Internal Medicine (IV), Osaka Medical and Pharmaceutical University, Osaka 569-1094, Japan; 3 Research & Development Center, Department of Medical Statistics, Osaka Medical and Pharmaceutical University, Osaka 569-1094, Japan

**Keywords:** biomarker, macrophage, microscopic polyangiitis, peripheral neuropathy, tissue inhibitor of metalloproteinase

## Abstract

This study aimed to elucidate the pathomechanism of peripheral neuropathy (PN) in microscopic polyangiitis (MPA) and to identify biomarkers useful for diagnosis and severity assessment. Patients with MPA (*n* = 37) and other non-inflammatory neurological diseases (ONDs; *n* = 12) were enrolled, and the peripheral nerves of all patients were evaluated using nerve conduction studies. We compared the clinical characteristics and 14 serum biomarker profiles among patients with MPA and PN, MPA without PN, and ONDs. Patients with MPA had a higher prevalence of motor neuropathy than patients with ONDs. Among the patients with MPA, those with motor neuropathy had significantly higher total Birmingham Vasculitis Activity Scores and serum levels of C-reactive protein (CRP), tissue inhibitor of metalloproteinase-1 (TIMP-1), and interleukin-6 than patients without motor neuropathy. Multivariable analyses adjusted for age, serum CRP level, and diabetes mellitus showed that high serum levels of TIMP-1 were independently related to a diagnosis of motor neuropathy in MPA. Additionally, there were significant negative correlations between the serum levels of TIMP-1 and compound muscle action potential amplitudes. Serum levels of TIMP-1 may be associated with the pathomechanism of motor neuropathy in MPA and could be a useful biomarker for diagnosing and evaluating the severity of motor neuropathy in MPA.

## 1. Introduction

Microscopic polyangiitis (MPA) is a subtype of antineutrophil cytoplasmic antibody (ANCA)-associated vasculitis (AAV) and was classified as necrotizing vasculitis affecting small vessels at the 2012 International Chapel Hill Consensus Conference [1]. MPA has been observed in 50% of Japanese patients with AAV and involves several organ systems, including the renal, respiratory, and nervous systems [1,2]. Myeloperoxidase (MPO) and proteinase-3 (PR-3) are the autoantigens for ANCA, and MPO-ANCA is detectable in 97.4% of patients with MPA in Japan [2]. ANCA activates neutrophils, leading to the activation of other inflammatory cells, such as B cells, T cells, and macrophages [3,4]. These processes induce the production of inflammatory cytokines in these cells. This ANCA-cytokine sequence leads to vascular inflammation and necrosis in AAV [5,6].

Peripheral neuropathy is reported to occur in approximately 45.0% of MPA cases [7]. A previous report showed that 29% of MPA patients have pure sensory neuropathy, and 6% have pure motor neuropathy [8]. Early immunosuppressive interventions are needed for progressive vasculitic neuropathy because it causes long-term sequelae [7,9,10]. However, 7.8% of patients in the early phase of vasculitic neuropathy were asymptomatic [11], and this may lead to the underestimation of peripheral neuropathy in MPA. Peripheral nerve biopsy is useful for the diagnosis of vasculitic neuropathy [12], but a biopsy is invasive and not suitable for all patients with vasculitic neuropathy. Nerve conduction studies (NCSs) are frequently used for evaluating the peripheral nervous system [13]. The compound muscle action potential (CMAP) and the sensory nerve action potential (SNAP) are obtained by electrically stimulating the motor nerve fibers and the sensory nerve fibers, respectively [13]. CMAP and SNAP amplitudes are measured to evaluate the severity of motor neuropathy and sensory neuropathy, respectively [14].

Traditional serum biomarkers, such as C-reactive protein (CRP) and MPO-ANCA, cannot predict the presence and severity of peripheral motor neuropathy and sensory neuropathy in MPA [8]. Therefore, new biomarkers are needed for diagnosing and assessing the severity of peripheral motor neuropathy and sensory neuropathy.

The etiology of peripheral neuropathy in MPA is the ischemic occlusion of the vasa nervorum, leading to an axonal degeneration of the peripheral nerve [9,15,16]. CD68-positive macrophages, neutrophils, B cells, and T cells are associated with the pathomechanism in vasculitic neuropathy [17,18,19]. These cells produce inflammatory cytokines and proteinases, such as tumor necrosis factor (TNF)-α, interleukin (IL)-1β, IL-6, and matrix metalloproteinase (MMP)-9, leading to nerve injury in vasculitic neuropathy [20,21,22,23]. However, the pathomechanism of peripheral neuropathy in MPA has not yet been elucidated. This study aimed to investigate candidate serum biomarkers for diagnosing peripheral motor neuropathy and sensory neuropathy in MPA. We performed a comprehensive analysis of serum biomarkers in MPA and investigated the association between biomarkers and the severity of peripheral motor neuropathy and sensory neuropathy.

## 2. Results

### 2.1. Clinical Characteristics of Patients with MPA

Thirty-seven patients with MPA were included in the present study, and the baseline clinical characteristics and disease severity classifications are presented in Table 1. The median age was 74.0 (69.5–79.5) years, and 18 patients (48.6%) were male. MPO-ANCA was positive in 35 (94.6%) patients, and PR-3-ANCA was positive in 3 (8.1%) patients. One patient was double positive for MPO-ANCA and PR-3-ANCA. The median total Birmingham Vasculitis Activity Score (BVAS) at onset was 18.0 (11.0–23.0), and the proportions of the 2009 five-factor scores (FFS) of ≤1, =2, and ≥3 were 24.3%, 56.8%, and 18.9%, respectively. The proportions of “localized,” “early systemic,” “systemic,” and “severe” as defined by the European Vasculitis Study Group (EUVAS) classification were 2.7%, 16.2%, 67.6%, and 13.5%, respectively. Twenty-seven patients with MPA (73.0%) had sensory neuropathy, as evaluated by NCS, and 26 patients with MPA (70.3%) had motor neuropathy. The prevalence of systemic organ involvements is shown in Supplementary Table S1.

### 2.2. Comparison of Clinical Characteristics and Biomarker Levels between Patients with MPA and Patients with other Non-Inflammatory Neurological Diseases

A comparison of clinical characteristics between patients with MPA and patients with other non-inflammatory neurological diseases (ONDs) is presented in Table 2. There were no significant differences between patients with MPA and those with ONDs in terms of age, sex, and diabetes mellitus (DM). The serum levels of white blood cell (WBC), lactate dehydrogenase (LD), and CRP were significantly higher in patients with MPA than in patients with ONDs (*p* = 0.0004, 0.027, and <0.0001, respectively). The serum levels of hemoglobin (Hb) and albumin (Alb) were significantly lower in patients with MPA than in those with ONDs (*p* < 0.0001 and <0.0001, respectively). Four of 12 patients with ONDs had motor neuropathy; one had corticobasal syndrome, two had spinocerebellar degeneration, and one had dementia with Lewy bodies. In the ONDs group with motor neuropathy, the median age was 65.0 (51.8–78.3) years, and two patients (50%) had DM. The prevalence of peripheral motor neuropathy was significantly higher in patients with MPA than in those with ONDs (*p* = 0.039).

A comparison of the cytokine levels between patients with MPA and patients with ONDs is shown in Table 3. The serum levels of all cytokines were significantly higher in patients with MPA than in those with ONDs.

### 2.3. Comparison of Clinical Characteristics between Patients with MPA with and without Motor Neuropathy

The comparison of clinical characteristics between patients with MPA with and without motor neuropathy is presented in Table 4. The median age tended to be higher in patients with MPA with motor neuropathy than in those without motor neuropathy (*p* = 0.081). There were no significant differences in terms of sex and DM between the two groups. The serum levels of CRP and the total BVAS were significantly higher in patients with MPA with motor neuropathy than in those without motor neuropathy (*p* = 0.016 and 0.021, respectively). The serum levels of Alb were significantly lower in patients with MPA with motor neuropathy than in those without motor neuropathy (*p* = 0.013). The percentage of patients with “early systemic” MPA as defined by the EUVAS classification was significantly higher in patients with MPA without motor neuropathy than in those with motor neuropathy (*p* = 0.0054). A comparison of systemic organ involvements between patients with MPA with and without motor neuropathy is shown in Supplementary Table S2.

### 2.4. Comparison of Serum Biomarker Levels between Patients with MPA with and without Motor Neuropathy

**Figure 1:**
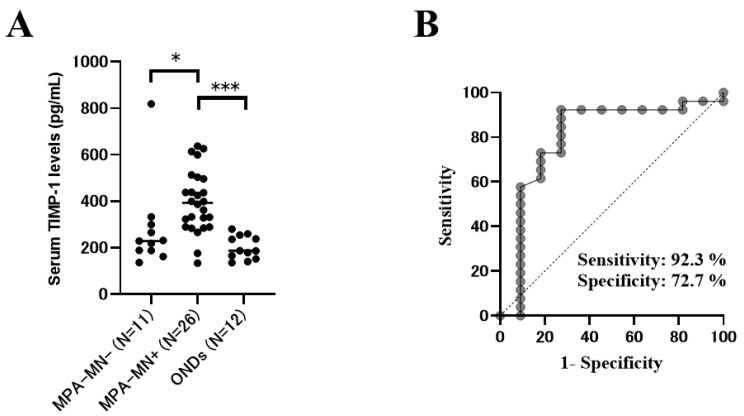
Functions of serum tissue inhibitor of metalloproteinase-1 (TIMP-1) levels as a biomarker for the diagnosis of microscopic polyangiitis (MPA)–motor neuropathy (MN). (**A**) Serum levels of TIMP-1 in the MPA-MN group, MPA without MN group, and other non-inflammatory neurological diseases (ONDs) group. (**B**) Area under the receiving operating characteristic curves for serum TIMP-1 levels to discriminate between MPA-MN and MPA without MN. The Mann–Whitney U test was used for the comparison of median values. A *p*-value of <0.05 was considered significant. * *p* < 0.05, *** *p* < 0.001.

A comparison of the cytokine levels between patients with MPA with and without motor neuropathy is shown in Table 5. In a univariate analysis, the serum levels of IL-6 and tissue inhibitor of metalloproteinase (TIMP)-1 were significantly higher in patients with MPA with motor neuropathy than in those without motor neuropathy (*p* = 0.039 and 0.0047, respectively) (Figure 1A). The serum levels of MMP-9 tended to be higher in patients with MPA with motor neuropathy than in those without motor neuropathy; however, the difference was not significant (*p* = 0.056).

### 2.5. Association of Biomarkers in MPA with Motor Neuropathy in Multivariable Analyses

To estimate the cutoff points for diagnosing motor neuropathy in MPA, the receiver operating characteristic (ROC) curves were generated using the serum levels of TIMP-1 and IL-6, revealing the optimal cutoff points of 266.2 pg/mL (area under the ROC curve (AUC), 0.80; sensitivity, 92.3%; specificity, 72.7%) and 26.6 pg/mL (AUC, 0.72; sensitivity, 65.4%; specificity, 81.8%), respectively (Figure 1B). The best AUCs were observed for TIMP-1 in these biomarkers.

To evaluate whether high serum TIMP-1 levels were independently associated with the diagnosis of motor neuropathy in MPA, we compared the serum TIMP-1 levels between the patients with MPA with motor neuropathy and those without motor neuropathy using multivariable analysis. We selected the covariates as age, CRP levels, and DM, based on the univariate analysis and a previous study [24], and we conducted a multivariable analysis. 

Table 6 shows the results of the multivariable analysis adjusted for age, CRP level, and DM. High serum levels of TIMP-1 were independently related to the diagnosis of motor neuropathy in MPA (*p* = 0.021).

### 2.6. Correlations between Serum Tissue Inhibitor of Metalloproteinase-1 Level and Compound Muscle Action Potential Amplitude

**Figure 2:**
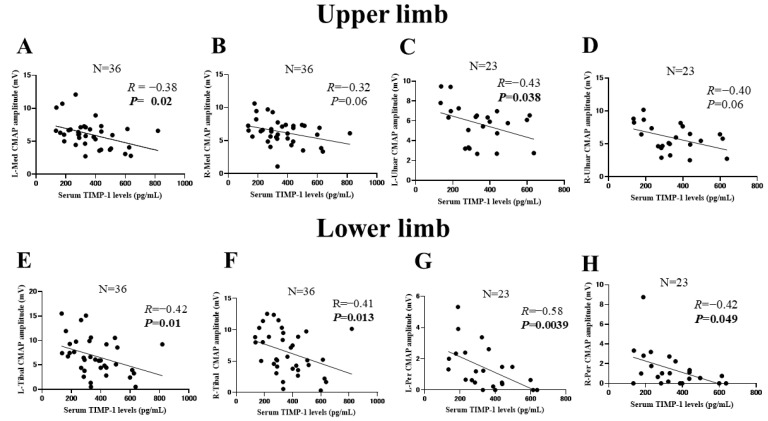
Correlations between serum tissue inhibitor of metalloproteinase-1 (TIMP-1) levels and compound muscle action potential (CMAP) amplitudes of the upper and lower limbs. (**A**–**D**) Correlations between serum TIMP-1 levels and CMAP amplitudes of the upper limbs: left median nerve (*n* = 36, (**A**)), right median nerve (*n* = 36, (**B**)), left ulnar nerve (*n* = 23, (**C**)), right ulnar nerve (*n* = 23, (**D**)). (**E**–**H**) Correlations between serum TIMP-1 levels and CMAP amplitudes of the lower limbs: left tibial nerve (*n* = 36, (**E**)), right tibial nerve (*n* = 36, (**F**)), left peroneal nerve (*n* = 23, (**G**)), right peroneal nerve (*n* = 23, (**H**)). Correlations were evaluated using Spearman’s correlation coefficients. A *p*-value of <0.05 was considered significant. L: left; R: Right; Med: median; Per: peroneal.

To evaluate whether the serum TIMP-1 levels were a useful biomarker for the severity of motor neuropathy in MPA, the correlations between the serum TIMP-1 levels and CMAP amplitudes were examined (Figure 2). The serum levels of TIMP-1 were significantly negatively correlated with the CMAP amplitudes of the left median nerve (R = −0.38), left ulnar nerve (R = −0.43), left tibial nerve (R = −0.42), right tibial nerve (R = −0.41), left peroneal nerve (R = −0.58), and right peroneal nerve (R = −0.42) (Figure 2A,C,E–H).

### 2.7. Correlations between Serum Tissue Inhibitor of Metalloproteinase-1 Level and Disease Activity in MPA

Next, we also examined the correlations between the serum TIMP-1 levels and systemic disease activity indicators, including total BVAS, Alb, and CRP (Supplementary Figure S1). The serum levels of TIMP-1 tended to be positively correlated with the total BVAS (R = 0.32) (Supplementary Figure S1A), significantly positively correlated with the CRP (R = 0.79) (Supplementary Figure S1B), and significantly negatively correlated with the serum Alb (R = −0.66) (Supplementary Figure S1C).

### 2.8. Correlations between Serum Tissue Inhibitor of Metalloproteinase-1 Level and Compound Muscle Action Potential Amplitudes after Immunosuppressive Therapy

To examine whether the initial serum TIMP-1 levels were a useful biomarker for predicting the prognosis of motor neuropathy in MPA, we evaluated the correlations between the initial serum TIMP-1 levels and CMAP amplitudes after immunosuppressive therapy (Supplementary Figure S2A–H). Twelve patients with MPA repeatedly conducted NCSs after immunosuppressive therapy. The mean number of follow-up days of the NCSs was 152 (48–1051) days. Out of 25 patients who could not repeatedly conduct NCS, 16 patients were excluded during follow-up because 9 patients were dead, and 7 patients were moved to other hospitals. The initial serum TIMP-1 levels were significantly negatively correlated with the CMAP amplitudes of the left tibial nerve (R = −0.72) and right tibial nerve (R = −0.65) (Supplementary Figure S2E,F). These findings suggest that initial serum TIMP-1 levels are useful biomarkers for predicting the severity of motor neuropathy after immunosuppressive therapy in patients with MPA.

### 2.9. Comparison of Clinical Characteristics and Biomarker Levels between Patients with MPA with and without Sensory Neuropathy

Comparisons of the clinical characteristics and serum biomarker levels between patients with MPA with and without sensory neuropathy are presented in Table 7 and Table 8, respectively. There were no significant differences between the patients with MPA with and without sensory neuropathy in terms of age, sex, DM, or laboratory findings. The percentage of patients with FFS ≤ 1 was significantly higher in patients with MPA without sensory neuropathy than in those with sensory neuropathy (*p* = 0.041). A comparison of systemic organ involvements between the patients with MPA with and without sensory neuropathy is shown in Supplementary Table S3.

The serum levels of IL-6 and IL-8 tended to be higher in patients with MPA with sensory neuropathy than in those without sensory neuropathy; however, this difference was not statistically significant (*p* = 0.19 and 0.12, respectively).

## 3. Discussion

The present study revealed that the prevalence of motor neuropathy and the serum levels of cytokines related to inflammatory cells (T cells, macrophages, B cells, and neutrophils) in patients with MPA were higher than those in patients with ONDs. Systemic inflammation, disease activity, and the serum levels of TIMP-1 and IL-6 in patients with MPA with motor neuropathy were significantly higher than those in patients with MPA without motor neuropathy. The multivariable analysis adjusted for age, CRP level, and DM showed that high serum levels of TIMP-1 were independently related to the diagnosis of motor neuropathy in MPA. There were significant negative correlations between the serum levels of TIMP-1 and CMAP amplitudes in the lower limbs, suggesting that the serum levels of TIMP-1 were associated with the severity of axonal involvement. In addition, the serum levels of IL-6 and IL-8 in patients with MPA with sensory neuropathy tended to be higher than those in patients with MPA without sensory neuropathy.

TIMP-1 is a selective inhibitor of MMP-9 and specifically interacts with proMMP-9 [25,26]. TIMP-1 is expressed in many tissues and increases concomitantly with the elevation of MMP levels [27,28,29,30]. TIMP-1 forms MMP-TIMP complexes and specifically inhibits the activation and functions of MMP-9 [25,31]. Several reports have shown the clinical utility of serum TIMP-1 in AAV. It has been reported that serum TIMP-1 was a useful marker for distinguishing active AAV from remission [32], and a predictive marker of sustained remission in AAV [30]. In addition, serum TIMP-1 levels are positively correlated with disease severities, such as CRP and total BVAS [32,33]. However, the association between TIMP-1 and the etiology and organ lesions in patients with MPA remains unclear. Ishizaki et al. reported that serum TIMP-1 levels were not associated with specific organ involvements, such as kidney and lungs, but the correlation between peripheral neuropathy and serum TIMP-1 levels was not examined [33]. In the present study, we first showed that the serum levels of TIMP-1 and MMP-9 were higher in patients with MPA with motor neuropathy than in those without motor neuropathy. In addition, we revealed that the serum levels of TIMP-1 were positively correlated with the severity of motor neuropathy in MPA.

TIMP-1 and MMP-9 were reportedly associated with the etiology of vasculitic neuropathy. MMP-9 is secreted from various cells, including macrophages, neutrophils, and fibroblasts, and is upregulated by proinflammatory cytokines, such as TNF-α and IL-1β [34,35]. In nerve biopsies of vasculitic neuropathy, MMP-9 is upregulated in blood vessel walls, the epineurium, and the endoneurium [16,23]. MMP-9 promotes the degradation of the blood-nerve barrier, leading to the invasion of macrophages into the damaged nerves [23]. Macrophages producing inflammatory cytokines promoted the expression of TIMP-1 in the transected sciatic nerves of mice [36]. Previous reports have shown that damage to motor neurons causes the upregulation of TIMP-1 secretion [37,38]. It has been reported that the TIMP-1 levels were elevated in serum and cerebrospinal fluid samples of amyotrophic lateral sclerosis, which causes a progressive degeneration of motor neurons [37,38]. These reports support the results of the present study, and the TIMP-1/MMP-9 axis might be related to the pathomechanism of motor neuropathy in patients with MPA. In injured sciatic nerves of mice, the antibody against MMP-9 significantly inhibited the invasion of macrophages into the damaged nerves [39]. In addition, TIMP-1 has a potential role in protecting the basement membrane after nerve injury [36]. These reports suggest that TIMP-1 might be protectively associated with motor neurons in patients with MPA.

NCSs are an essential tool for diagnosing peripheral neuropathy. However, the peripheral neuropathy of patients with external cardiac pacing wires and intracardiac catheters should not be evaluated using NCSs because surface nerve stimulation causes a significant risk of electrical injury to the heart [13,40]. For such patients, measurement of the serum TIMP-1 may be helpful for diagnosing peripheral neuropathy. In addition, in facilities where NCSs cannot be performed or where a neurologist is absent, measurement of the serum TIMP-1 may be useful for diagnosing peripheral neuropathy. In addition, serum TIMP-1 may be useful for the adjunctive diagnosis of motor neuropathy in MPA because serum TIMP-1 is correlated with the disease severity of motor neuropathy and the predicted prognosis of motor neuropathy in MPA (Figure 2 and Supplementary Figure S2).

In the Diagnostic and Classification Criteria for Vasculitis study, patients with MPA with peripheral neuropathy had a significantly lower prevalence of renal and gastrointestinal involvement than patients with MPA without peripheral neuropathy [8]. In the present study, the total BVAS score was significantly higher in patients with MPA with motor neuropathy than in those without motor neuropathy. This finding suggests an association between systemic disease activity and motor neuropathy in MPA.

In the present study, there was no significant difference in the prevalence of sensory neuropathy between patients with MPA and those with ONDs. Since the patients with MPA were older, age might have affected this result as a confounding factor [41]. In addition, in this study, there were no patients who were diagnosed with diabetic neuropathy, but DM might affect this result because patients with DM may have latent sensory nerve damage [24]. The serum levels of IL-6 and IL-8 tended to be higher in patients with MPA with sensory neuropathy than in those without sensory neuropathy. Previous reports have shown that CD68-positive macrophages were activated on the sural nerves of patients with MPA with peripheral neuropathy [17], and IL-6 was overexpressed by CD68-positive macrophages in sural nerve specimens of vasculitic neuropathy [20]. These previous reports support our results, and macrophages might be associated with sensory neuropathy in patients with MPA. Our results also suggest that neutrophils may be related to sensory neuropathy in patients with MPA by producing IL-8.

The present study has some limitations. First, all patients were Japanese, and the rate of positive MPO-ANCA was higher than that in other ethnicities. It is necessary to validate whether serum TIMP-1 levels are useful biomarkers for patients with MPA of other ethnicities. Second, we could not evaluate all nerves in patients with MPA; thus, the prevalence of neuropathy might have been underestimated. Third, there was no significance in the prevalence of renal involvement between MPA with and without motor neuropathy. However, a previous report showed associations between renal impairment and peripheral neuropathy [42], and we cannot deny the influence of renal involvement on motor neuropathy in MPA. Therefore, further investigations are needed to elucidate the pathomechanism of motor neuropathy of MPA by examining the patients without renal involvement. Fourth, we adopted the standard values used in studies targeting Japanese subjects. However, it remains unknown whether the standard values are applicable to other ethnicities because ethnic differences in amplitude measurements in nerve conduction studies have been reported [43]. Further investigations were needed by using widely accepted normative data, such as the American Association of Neuromuscular & Electrodiagnostic Medicine, to elucidate useful biomarkers for the peripheral neuropathy of MPA in other ethnicities. In addition, future studies are needed to further characterize the pattern of neuropathy (axonal/demyelinating peripheral polyneuropathy versus mononeuritis multiplex) by nerve conduction and needle EMG studies and the correlation with biomarkers. Finally, our study was a retrospective observational study involving a small number of patients in a single institution. Therefore, we might overestimate the value of serum TIMP-1 levels as biomarkers of motor neuropathy in MPA. Therefore, further investigations in large populations are needed to evaluate whether serum TIMP-1 levels are useful biomarkers for diagnosing and predicting the severity of motor neuropathy in MPA.

## 4. Materials and Methods

### 4.1. Patients with MPA

We investigated patients who were newly diagnosed with MPA at Osaka Medical and Pharmaceutical University Hospital between September 2011 and April 2019. The diagnosis of MPA was based on the Chapel Hill Consensus definition revised in 2012 [1]. In the diagnosis of MPA, patients with infection, drug reaction, malignancy, sarcoidosis, secondary vasculitis, and vasculitis mimics were excluded [44]. Patients with MPA whose peripheral nerves were evaluated in an NCS were enrolled in this study. Patients with other known causes of neuropathy, including endocrine disorders, vitamin deficiency, toxic exposure, alcoholism, paraneoplasm, and other autoimmune diseases, were excluded [41]. There were no patients with MPA who were diagnosed with diabetic neuropathy on admission. All patients were admitted to our hospital for the first remission induction therapy. All patients received immunosuppressive treatments at the physician’s discretion.

All clinical data and laboratory findings were obtained from medical records. The present study was conducted in accordance with the Declaration of Helsinki and its amendments and was approved by the Osaka Medical and Pharmaceutical University and the Faculty of Medicine Ethics Committee (approval no. 1529). Informed consent was obtained from all patients.

### 4.2. Patients with Other Non-Inflammatory Neurological Diseases

To compare the clinical and laboratory findings between MPA and non-MPA, we examined 12 patients with ONDs who were serially admitted to our hospital between February 2018 and April 2021 [45,46]. Their peripheral nerves were evaluated using an NCS. The ONDs included the following disorders: spinocerebellar degeneration in 8 (including multiple system atrophy), Parkinson’s disease in 1, dementia with Lewy bodies in 1, corticobasal syndrome in 1, and myoclonus in 1. These diseases are not typically accompanied by motor and sensory neuropathy due to immunological mechanisms [47]. NCSs cannot be performed in healthy controls because it is invasive [48], so we used an ONDs group as a control group to examine the relationship between the inflammatory response and peripheral neuropathy of MPA in this study [47]. We repeatedly collected the data of NCSs, and the last follow-up period was in November 2020.

### 4.3. Clinical Assessments

The patient clinical characteristics (age, sex, and DM) were evaluated. The WBC counts, Hb, Alb, LD, creatinine, CRP, and HbA1c levels were measured. Serum MPO-ANCA and PR-3-ANCA titers were measured using an enzyme-linked immunosorbent assay (ELISA) commercially conducted by SRL (SRL Inc., Tokyo, Japan).

### 4.4. Measurements of Serum Biomarkers

To measure the biomarkers, sera were collected before immunosuppressive therapy and stored at −70 °C until analysis. We measured the serum levels of the biomarkers, including IL-1β, IL-2, IL-4, IL-6, IL-8, IL-10, IL-13, TNF-α, interferon-γ, granulocyte colony-stimulating factor (G-CSF), and macrophage (M)-CSF, using a cytometric bead array method (LXSAHM R&D Human Luminex Screening Assay, Minneapolis, MN, USA). The serum levels of TIMP-1, MMP-9, and transforming growth factor-β were measured using ELISA kits (R&D Systems, Cambridge, UK).

### 4.5. Assessment of Peripheral Nerves

The peripheral nerves were evaluated by NCSs in the median, ulnar, tibial, peroneal, and sural nerves using the standard techniques of surface stimulation and recording [49]. We evaluated the peripheral nerves using a Neuropack X1 MEB-2306 (Nihon Kouden, Tokyo, Japan). Peripheral neuropathy was diagnosed based on electrophysiological signs. SNAP and CMAP amplitudes were measured for assessing the severity of sensory neuropathy and motor neuropathy, respectively. Patients with MPA with decreased amplitudes of nerve action potentials in one or more nerves in NCS were defined as the peripheral neuropathy group. The definitions of decreased amplitudes for sensory and motor nerve action potentials were as follows: SNAP amplitude (baseline to negative peak) (median nerve < 13.86 μV, ulnar nerve < 10.77 μV, sural nerve < 5.00 μV) and CMAP amplitude (baseline to negative peak) (median nerve < 3.95 mV, ulnar nerve < 4.22 mV, tibial nerve < 7.28 mV, peroneal nerve < 0.6 mV) [14,50].

### 4.6. Evaluation of Disease Severity

BVAS version 3 was used to evaluate organ involvements [51]. The 2009 FFS, which is the poor prognosis indicator of AAV, was calculated for each patient [52]. The EUVAS categorization system was used to evaluate disease severity [53].

### 4.7. Statistical Analyses

Categorical variables are expressed as numbers and percentages. Continuous variables are presented as medians and interquartile ranges. Fisher’s exact test was used to compare the categorical variables, and Wilcoxon’s rank-sum test was used to compare the continuous variables. The accuracy of each serum biomarker for diagnosing motor neuropathy was assessed using an ROC curve analysis. High levels of biomarkers were calculated from the ROC curves obtained for diagnosing motor neuropathy in patients with MPA. 

Because of the relatively few motor neuropathy cases, we used a propensity score adjustment for the serum TIMP-1 in the multivariable analysis, which was in accordance with a previous study [54]. The propensity score adjustment preserved statistical power by reducing the covariates into a single variable [54]. When the adjusted effect of motor neuropathy was evaluated, the propensity score was created through a binary logistic regression providing the predicted probability of motor neuropathy as a function of the other risk factors (age, CRP, and DM). The propensity score was computed for the serum TIMP-1 levels and then used as a covariate in the model. Multivariable analysis was adjusted for age, CRP level, and the presence of DM using a propensity score adjustment, as previously described [54,55]. Correlations were evaluated using Spearman’s correlation coefficient. Two-sided *p*-values less than 0.05 were considered statistically significant. The confidence interval was 95%. All data analyses were performed using JMP^®^ Pro version 15 (SAS Institute Inc., Cary, NC, USA) and GraphPad Prism (GraphPad Software, La Jolla, CA, USA).

## 5. Conclusions

TIMP-1 might be associated with the pathomechanism of motor neuropathy in MPA. The serum levels of TIMP-1 might be a useful biomarker for diagnosing and evaluating the severity of motor neuropathy in MPA. Further investigations are needed to reveal the associations between serum cytokines and peripheral neuropathy in patients with MPA.

## Figures and Tables

**Table 1:** Clinical characteristics of patients with MPA.

Characteristics	MPA (*n* = 37)
Age, years	74.0 (69.5–79.5)
Male, *n* (%)	18 (48.6)
Diabetes mellitus, *n* (%)	10 (27.0)
Systemic symptoms	
Cutaneous, *n* (%)	2 (5.4)
Chest, *n* (%)	5 (13.5)
Renal, *n* (%)	27 (73.0)
Laboratory findings	
WBC, ×10^3^/mm^3^	12.9 (8.6–15.8)
Hb, g/dL	10.0 (8.6–11.8)
Alb, g/dL	2.4 (2.0–3.0)
LD, IU/L	197.0 (166.0–232.0)
Cr, mg/dL	1.2 (0.76–1.7)
CRP, mg/mL	9.6 (2.9–12.6)
Positive, anti-MPO-ANCA, *n* (%)	35 (94.6)
Positive, anti-PR3-ANCA, *n* (%)	3 (8.1)
MPO-ANCA titer, U/mL	97.4 (71.4–274.0)
HbA1c, %	5.9 (5.6–6.3) ^a^
BVAS at onset	18.0 (11.0–23.0)
Five-factor score 2009	
≤1, *n* (%)	9 (24.3)
2, *n* (%)	21 (56.8)
≥3, *n* (%)	7 (18.9)
EUVAS-defined disease activity	
Localized, *n* (%)	1 (2.7)
Early systemic, *n* (%)	6 (16.2)
Systemic, *n* (%)	25 (67.6)
Severe, *n* (%)	5 (13.5)
Peripheral neuropathy	
Sensory neuropathy, *n* (%)	27 (73.0)
Motor neuropathy, *n* (%)	26 (70.3)

Categorical variables are presented as a number (%). Continuous variables are presented as the median (interquartile range). MPA: microscopic polyangiitis; WBC: white blood cell; Hb: hemoglobin; Alb: albumin; LD: lactate dehydrogenase; Cr: creatinine; CRP: C-reactive protein; MPO-ANCA: myeloperoxidase-anti-neutrophil cytoplasmic autoantibody; PR3-ANCA: proteinase 3-anti-neutrophil cytoplasmic antibody; BVAS: Birmingham Vasculitis Activity Score; EUVAS: European Vasculitis Study Group. ^a^ Number of subjects, *n* = 35.

**Table 2:** Comparison of clinical characteristics between patients with MPA and patients with ONDs.

Characteristics	MPA (*n* = 37)	ONDs (*n* = 12)	*p*-Value
Age, years	74.0 (69.5–79.5)	72.5 (58.8–78.3)	0.29
Male, *n* (%)	18 (48.6)	6 (50.0)	1.00
Body weight, kg	58.0 (47.0–64.2)	51.7 (44.9–63.2)	0.41
Diabetes mellitus, *n* (%)	10 (27.0)	3 (25.0)	1.00
Laboratory findings			
WBC, ×10^3^/mm^3^	12.9 (8.6–15.8)	6.4 (4.9–7.8)	0.0004 *
Hb, g/dL	10.0 (8.6–11.8)	13.2 (11.7–14.5)	<0.0001 *
Alb, g/dL	2.4 (2.0–3.0)	3.9 (3.6–4.2)	<0.0001 *
LD, IU/L	197.0 (166.0–232.0)	171.0 (158.0–193.0)	0.027 *
Cr, mg/dL	1.2 (0.76–1.7)	0.89 (0.64–1.1)	0.063
CRP, mg/mL	9.6 (2.9–12.6)	0.040 (0.015–0.28)	<0.0001 *
HbA1c, %	5.9 (5.6–6.3) ^a^	5.7 (5.5–6.1)	0.49
Peripheral neuropathy			
Sensory neuropathy, *n* (%)	27 (73.0)	5 (41.7)	0.080
Motor neuropathy, *n* (%)	26 (70.3)	4 (33.3)	0.039 *

Categorical variables are presented as a number (%). Continuous variables are presented as the median (interquartile range). The *p*-values were estimated using Fisher’s exact test or Wilcoxon rank sum test. * *p* < 0.05. MPA: microscopic polyangiitis; ONDs: other non-inflammatory neurological diseases; WBC: white blood cell; Hb: hemoglobin; Alb: albumin; LD: lactate dehydrogenase; Cr: creatinine; CRP: C-reactive protein. ^a^ Number of subjects, *n* = 35.

**Table 3:** Comparison of the cytokine levels between patients with MPA and patients with ONDs.

Cytokines	MPA (*n* = 37)	ONDs (*n* = 12)	*p*-Value
Th1-related cytokine			
IL-2, pg/mL	5.7 (4.1–7.8)	1.0 (1.0–1.0)	0.0004 *
IFN-γ, pg/mL	21.6 (11.2–36.4)	0.63 (0.63–0.63)	<0.0001 *
Th2-related cytokine			
IL-4, pg/mL	93.3 (83.0–99.9)	15.7 (15.7–15.7)	<0.0001 *
IL-13, pg/mL	403.6 (265.9–519.9)	92.0 (92.0–92.0)	<0.0001 *
M1 Macrophage-related cytokine			
IL-1β, pg/mL	5.0 (2.5–8.1)	1.5 (1.5–1.5)	<0.0001 *
TNF-α, pg/mL	13.0 (9.8–15.4)	3.7 (2.0–6.0)	<0.0001 *
M2 Macrophage-related cytokine			
IL-10, pg/mL	1.2 (0.17–2.5)	0.05 (0.05–0.1)	<0.0001 *
M-CSF, pg/mL	135.9 (56.7–388.8)	0.69 (0.69–0.69)	<0.0001 *
B cell-related cytokine			
IL-6, pg/mL	26.6 (11.5–71.2)	1.2 (1.2–3.4)	<0.0001 *
Neutrophil-related cytokine			
G-CSF, pg/mL	39.2 (26.6–54.2)	3.2 (3.2–6.1)	<0.0001 *
IL-8, pg/mL	27.9 (17.6–55.7)	6.3 (4.1–8.5)	0.0002 *
Pro-fibrotic biomarkers			
TIMP-1, pg/mL	331.1 (248.4–438.3)	186.7 (155.5–250.4)	0.0003 *
MMP-9, ng/mL	840.5 (379.3–1315.2)	191.0 (137.7–274.9)	<0.0001 *
TGF-β, ng/mL	36.7 (26.5–50.5)	6.6 (5.2–8.0)	<0.0001 *

Continuous variables are presented as the median (interquartile range). The *p*-values were estimated using Wilcoxon rank sum test. * *p* < 0.05. MPA: microscopic polyangiitis; ONDs: other non-inflammatory neurological diseases; IL: interleukin; IFN: interferon; TNF: tumor necrosis factor; M-CSF: macrophage colony-stimulating factor; G-CSF: granulocyte colony-stimulating factor; TIMP: tissue inhibitor of metalloproteinase; MMP: matrix metalloproteinase; TGF: transforming growth factor.

**Table 4:** Comparison of clinical characteristics between patients with MPA with and without motor neuropathy.

Characteristics	MPA with Motor Neuropathy (*n* = 26)	MPA without Motor Neuropathy (*n* = 11)	*p*-Value
Age, years	75.5 (71.8–80.5)	70.0 (66.0–78.0)	0.081
Male, *n* (%)	12 (46.2)	6 (54.6)	0.73
Diabetes mellitus, *n* (%)	7 (26.9)	3 (27.3)	1.00
Systemic Symptoms			
Cutaneous, *n* (%)	1 (3.9)	1 (9.1)	0.51
Chest, *n* (%)	4 (15.4)	1 (9.1)	1.00
Renal, *n* (%)	20 (76.9)	7 (63.6)	0.44
Laboratory findings			
WBC, ×10^3^/mm^3^	13.7 (10.1–16.9)	9.7 (6.7–13.6)	0.056
Hb, g/dL	9.6 (8.3–11.1)	10.7 (9.7–12.2)	0.15
Alb, g/dL	2.2 (1.9–2.7)	2.9 (2.3–3.3)	0.013 *
LD, IU/L	208.0 (166.0–253.5)	194.0 (165.0–210.0)	0.42
Cr, mg/dL	1.3 (0.78–1.9)	1.1 (0.61–1.3)	0.30
CRP, mg/mL	11.6 (3.7–13.6)	3.9 (0.61–9.5)	0.016 *
Positive, anti-MPO-ANCA, *n* (%)	24 (92.3)	11 (100.0)	1.00
Positive, anti-PR3-ANCA, *n* (%)	3 (11.5)	0 (0.0)	0.54
MPO-ANCA titer, U/mL	96.3 (72.6–267.5)	131.0 (68.0–295.0)	0.70
HbA1c, %	5.9 (5.7–6.3) ^a^	5.8 (5.5–6.3) ^b^	0.66
BVAS at onset	20.5 (14.0–25.0)	12.0 (8.0–18.0)	0.021 *
BVAS score excluding nervous system	14.5 (12–18.3)	12 (5–14)	0.067
Five-factor score 2009			
≤1, *n* (%)	4 (15.4)	5 (45.5)	0.091
2, *n* (%)	16 (61.5)	5 (45.5)	0.48
≥3, *n* (%)	6 (23.1)	1 (9.1)	0.65
EUVAS-defined disease activity			
Localized, *n* (%)	1 (3.9)	0 (0.0)	1.00
Early systemic, *n* (%)	1 (3.9)	5 (45.5)	0.0054 *
Systemic, *n* (%)	20 (76.9)	5 (45.5)	0.12
Severe, *n* (%)	4 (15.4)	1 (9.1)	1.00

Categorical variables are presented as a number (%). Continuous variables are presented as the median (interquartile range). The *p*-values were estimated using Fisher’s exact test or Wilcoxon rank sum test. * *p* < 0.05. MPA: microscopic polyangiitis; WBC: white blood cell; Hb: hemoglobin; Alb: albumin; LD: lactate dehydrogenase; Cr: creatinine; CRP: C-reactive protein; MPO-ANCA: myeloperoxidase-anti-neutrophil cytoplasmic autoantibody; PR3-ANCA: proteinase 3-anti-neutrophil cytoplasmic antibody; BVAS: Birmingham Vasculitis Activity Score; EUVAS: European Vasculitis Study Group. ^a^ Number of subjects, *n* = 25. ^b^ Number of subjects, *n* = 10.

**Table 5:** Comparison of the cytokine levels between patients with MPA with and without motor neuropathy.

Cytokines	MPA with Motor Neuropathy (*n* = 26)	MPA without Motor Neuropathy (*n* = 11)	*p*-Value
Th1-related cytokine			
IL-2, pg/mL	5.1 (4.3–7.8)	5.7 (2.5–7.8)	0.92
IFN-γ, pg/mL	24.1 (11.2–53.0)	16.5 (8.6–31.5)	0.27
Th2-related cytokine			
IL-4, pg/mL	92.5 (85.6–100.7)	93.3 (83.0–99.9)	0.91
IL-13, pg/mL	444.1 (235.8–519.9)	360.9 (265.9–444.1)	0.31
M1 Macrophage-related cytokine			
IL-1β, pg/mL	5.0 (3.4–8.5)	5.0 (1.5–8.1)	0.44
TNF-α, pg/mL	13.9 (10.5–15.4)	11.2 (7.7–13.6)	0.18
M2 Macrophage-related cytokine			
IL-10, pg/mL	1.6 (0.17–2.8)	0.38 (0.17–2.0)	0.20
M-CSF, pg/mL	163.9 (78.8–431.0)	117.2 (5.8–145.2)	0.090
B cell-related cytokine			
IL-6, pg/mL	41.3 (14.7–74.4)	13.8 (6.2–25.7)	0.039 *
Neutrophil-related cytokine			
G-CSF, pg/mL	41.4 (29.4–64.0)	39.2 (17.8–43.7)	0.29
IL-8, pg/mL	32.4 (20.0–69.1)	19.3 (16.4–32.0)	0.17
Pro-fibrotic biomarkers			
TIMP-1, pg/mL	393.1 (289.5–498.0)	228.7 (187.9–299.3)	0.0047 *
MMP-9, ng/mL	1018.1 (458.4–1477.8)	555.4 (258.4–920.2)	0.056
TGF-β, ng/mL	37.9 (28.8–50.2)	28.9 (20.8–52.4)	0.38

Continuous variables are presented as the median (interquartile range). The *p*-values were estimated using Wilcoxon rank sum test. * *p* < 0.05. MPA: microscopic polyangiitis; IL: interleukin; IFN: interferon; TNF: tumor necrosis factor; M-CSF: macrophage colony-stimulating factor; G-CSF: granulocyte colony-stimulating factor; TIMP: tissue inhibitor of metalloproteinase; MMP: matrix metalloproteinase; TGF: transforming growth factor.

**Table 6:** Association of biomarkers in MPA with motor neuropathy in multivariable analysis.

Variable	*p* Value	OR (95% CI)
High IL-6	NS	-
High TIMP-1	0.021 *	14.1 (1.5–134.8)

Variables identified as significant at *p* < 0.05 in univariate analysis were tested in multivariable analysis. Covariates: age, C-reactive protein, and diabetes mellitus. Cutoff values to define high levels of biomarkers were obtained from receiver operating characteristic curve. High serum biomarker levels were defined as follows: for IL-6, >26.6 pg/mL; for TIMP-1, >266.2 pg/mL. * *p* < 0.05. MPA: microscopic polyangiitis; IL: interleukin; TIMP: tissue inhibitor of metalloproteinase. OR: odds ratio; 95% CI: 95% confidence interval; NS: not significant.

**Table 7:** Comparison of clinical characteristics between patients with MPA with and without sensory neuropathy.

Characteristics	MPA with Sensory Neuropathy (*n* = 27)	MPA without Sensory Neuropathy (*n* = 10)	*p*-Value
Age, years	75.0 (71.0–80.0)	69.5 (66.0–79.0)	0.16
Male, *n* (%)	14 (51.9)	4 (40.0)	0.71
Diabetes mellitus, *n* (%)	8 (29.6)	2 (20.0)	0.69
Systemic symptoms			
Cutaneous, *n* (%)	2 (7.4)	0 (0.0)	1.00
Chest, *n* (%)	3 (11.1)	2 (20.0)	0.60
Renal, *n* (%)	21 (77.8)	6 (60.0)	0.41
Laboratory findings			
WBC, ×10^3^/mm^3^	12.9 (9.0–15.9)	13.2 (7.5–15.8)	0.90
Hb, g/dL	9.8 (8.5–11.6)	10.1 (9.3–12.4)	0.42
Alb, g/dL	2.4 (2.0–2.9)	2.4 (2.1–3.2)	0.54
LD, IU/L	184.0 (161.0–234.0)	208.0 (189.0–247.3)	0.16
Cr, mg/dL	1.3 (0.79–1.8)	0.88 (0.72–1.5)	0.29
CRP, mg/mL	11.6 (3.0–13.3)	6.5 (2.2–10.7)	0.25
Positive, anti-MPO-ANCA, *n* (%)	25 (92.6)	10 (100.0)	1.00
Positive, anti-PR3-ANCA, *n* (%)	3 (11.1)	0 (0.0)	0.55
MPO-ANCA titer, U/mL	95.2 (62.5–287.0)	114.2 (73.1–239.0)	0.96
HbA1c, %	5.9 (5.5–6.4) ^a^	5.8 (5.7–6.1)	0.84
BVAS at onset	19.0 (14.0–23.0)	12.5 (7.5–21.3)	0.19
Five-factor score 2009			
≤1, *n* (%)	4 (14.8)	5 (50.0)	0.041 *
2, *n* (%)	17 (63.0)	4 (40.0)	0.27
≥3, *n* (%)	6 (22.2)	1 (10.0)	0.65
EUVAS-defined disease activity			
Localized, *n* (%)	0 (0.0)	1 (10.0)	0.27
Early systemic, *n* (%)	3 (11.1)	3 (30.0)	0.31
Systemic, *n* (%)	19 (70.4)	6 (60.0)	0.70
Severe, *n* (%)	5 (18.5)	0 (0.0)	0.30

Categorical variables are presented as a number (%). Continuous variables are presented as the median (interquartile range). The *p*-values were estimated using Fisher’s exact test or Wilcoxon rank sum test. * *p* < 0.05. MPA: microscopic polyangiitis; WBC: white blood cell; Hb: hemoglobin; Alb: albumin; LD: lactate dehydrogenase; Cr: creatinine; CRP: C-reactive protein; MPO-ANCA: myeloperoxidase-anti-neutrophil cytoplasmic autoantibody; PR3-ANCA: proteinase 3-anti-neutrophil cytoplasmic antibody; BVAS: Birmingham Vasculitis Activity Score; EUVAS: European Vasculitis Study Group. ^a^ Number of subjects, *n* = 25.

**Table 8:** Comparison of the cytokine levels between patients with MPA with and without sensory neuropathy.

Cytokines	MPA with Sensory Neuropathy (*n* = 27)	MPA without Sensory Neuropathy (*n* = 10)	*p*-Value
Th1-related cytokine			
IL-2, pg/mL	4.6 (3.5–7.8)	6.2 (4.1–7.8)	0.56
IFN-γ, pg/mL	16.5 (11.2–50.7)	26.6 (14.5–36.4)	0.62
Th2-related cytokine			
IL-4, pg/mL	91.6 (83.0–99.9)	95.0 (88.2–110.9)	0.35
IL-13, pg/mL	444.1 (145.6–519.9)	403.6 (337.2–480.7)	0.85
M1 Macrophage-related cytokine			
IL-1β, pg/mL	5.0 (1.5–8.1)	6.6 (2.9–8.9)	0.52
TNF-α, pg/mL	13.0 (9.5–15.4)	13.0 (9.9–16.1)	0.93
M2 Macrophage-related cytokine			
IL-10, pg/mL	1.2 (0.17–2.9)	1.1 (0.17–2.1)	0.66
M-CSF, pg/mL	126.5 (42.8–426.3)	145.2 (89.3–339.5)	0.95
B cell-related cytokine			
IL-6, pg/mL	34.0 (12.6–71.7)	14.4 (7.5–61.3)	0.19
Neutrophil-related cytokine			
G-CSF, pg/mL	39.2 (29.4–52.2)	43.7 (15.9–59.1)	0.92
IL-8, pg/mL	32.0 (20.2–59.4)	19.3 (9.4–49.1)	0.12
Pro-fibrotic biomarkers			
TIMP-1, pg/mL	333.3 (283.5–437.9)	264.0 (188.8–454.9)	0.38
MMP-9, ng/mL	998.5 (374.3–1294.4)	558.1 (461.7–1815.3)	0.80
TGF-β, ng/mL	36.3 (24.9–43.0)	39.1 (27.9–54.2)	0.42

Continuous variables are presented as the median (interquartile range). The *p*-values were estimated using Wilcoxon rank sum test. MPA: microscopic polyangiitis; IL: interleukin; IFN: interferon; TNF: tumor necrosis factor; M-CSF: macrophage colony-stimulating factor; G-CSF: granulocyte colony-stimulating factor; TIMP: tissue inhibitor of metalloproteinase; MMP: matrix metalloproteinase; TGF: transforming growth factor.

## Data Availability

The data presented in this study are available upon request from the corresponding author.

## Supplementary Materials

The supporting information can be downloaded at: https://www.mdpi.com/article/10.3390/ijms232113374/s1.
