# Supplementary material for: Association between Serum Biomarkers and Peripheral Neuropathy in Microscopic Polyangiitis
Source: Int J Mol Sci. 2022 Nov 2;23(21):13374. doi: 10.3390/ijms232113374 (PMC9658745; doi:10.3390/ijms232113374)
Supplement: Supplementary file 1 [file ijms-23-13374-s001.zip › ijms-2001241-supplementary.pdf]

Table S1. Clinical characteristics of patients with MPA.

Characteristics	MPA (n = 37)
Systemic Symptoms	
General, n (%)	28 (75.7)
Cutaneous, n (%)	2 (5.4)
Mucous membrane, n (%)	3 (8.1)
Ear, nose, throat, n (%)	14 (37.8)
Chest, n (%)	5 (13.5)
Cardiovascular, n (%)	3 (8.1)
Abdominal, n (%)	1 (2.7)
Renal, n (%)	27 (73.0)
Nervous system, n (%)	24 (64.9)

Categorical variables are presented as number (%). MPA: microscopic polyangiitis.

Table S2. Comparison of clinical characteristics between patients with MPA with and without motor neuropathy.

Characteristics	MPA with Motor Neuropathy (n = 26)	MPA without Motor Neuropathy (n = 11)	*p*-Value
Systemic Symptoms			
General, n (%)	20 (76.9)	8 (72.7)	1.00
Cutaneous, n (%)	1 (3.9)	1 (9.1)	0.51
Mucous membrane, n (%)	1 (3.9)	2 (18.2)	0.21
Ear, nose, throat, n (%)	10 (38.5)	4 (36.4)	1.00
Chest, n (%)	4 (15.4)	1 (9.1)	1.00
Cardiovascular, n (%)	3 (11.5)	0 (0.0)	0.54
Abdominal, n (%)	1 (3.9)	0 (0.0)	1.00
Renal, n (%)	20 (76.9)	7 (63.6)	0.44

Categorical variables are presented as number (%). The *p*-values were estimated using Fisher's exact test. MPA: microscopic polyangiitis.

Table S3. Comparison of clinical characteristics between patients with MPA with and without motor neuropathy.

Characteristics	MPA with Sensory Neuropathy (n= 27)	MPA without Sensory Neuropathy (n=10)	*p*-Value
Systemic Symptoms			
General, n (%)	20 (74.1)	8 (80.0)	1.00
Cutaneous, n (%)	2 (7.4)	0 (0.0)	1.00
Mucous membrane, n (%)	1 (3.7)	2 (20.0)	0.17
Ear, nose, throat, n (%)	9 (33.3)	5 (50.0)	0.45
Chest, n (%)	3 (11.1)	2 (20.0)	0.60
Cardiovascular, n (%)	2 (7.4)	1 (10.0)	1.00
Abdominal, n (%)	1 (3.7)	0 (0.0)	1.00
Renal, n (%)	21 (77.8)	6 (60)	0.41

Categorical variables are presented as number (%). The *p*-values were estimated using Fisher's exact test. MPA: microscopic polyangiitis.

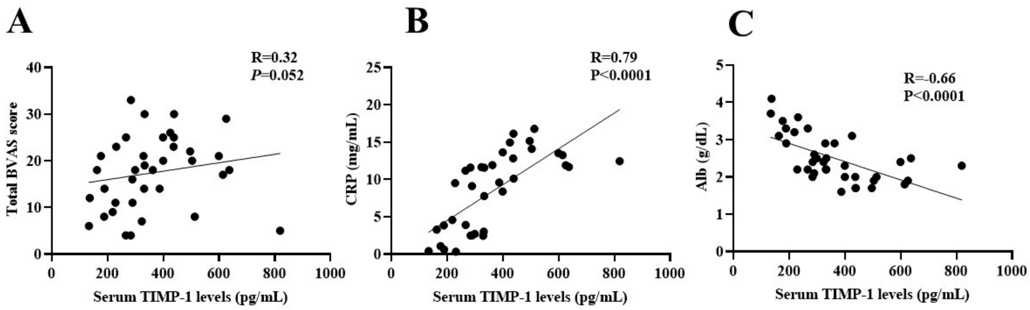

Figure S1. Correlations between serum tissue inhibitor of metalloproteinase-1 (TIMP-1) levels and systemic disease activity indicators in microscopic polyangiitis. (**A**) The correlation between serum TIMP-1 levels and total BVAS score. (**B**) The correlation between serum TIMP-1 levels and CRP. (**C**) The correlation between serum TIMP-1 levels and Alb. Correlations were evaluated using Spearman's correlation coefficients. A *p*-value of <0.05 was considered significant. BVAS: The Birmingham Vasculitis Activity Score, CRP: C-reactive protein, Alb: albumin.

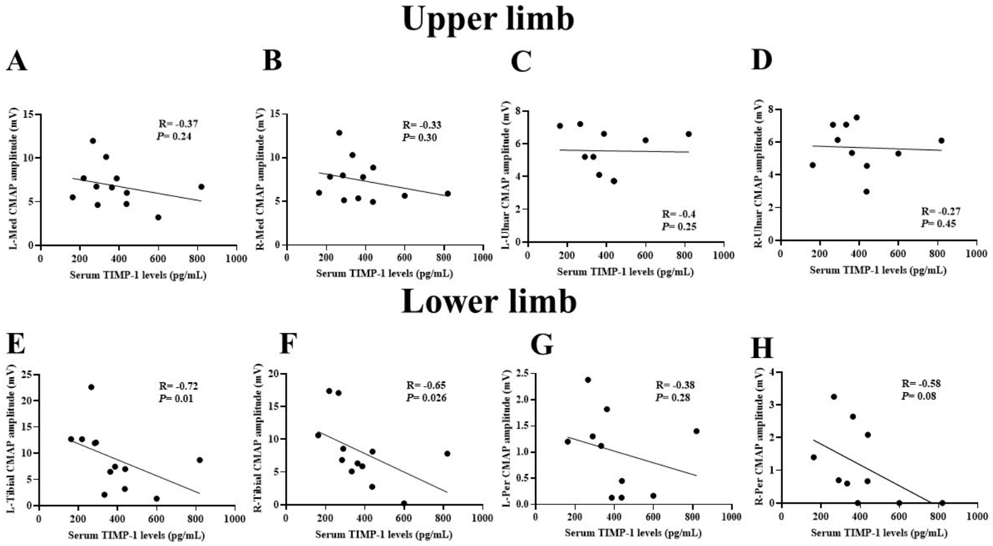

Figure S2. Correlations between serum tissue inhibitor of metalloproteinase-1 (TIMP-1) levels and compound muscle action potential (CMAP) amplitudes of the upper and lower limbs after immunosuppressive therapy. (**A**–**D**) Correlations between serum TIMP-1 levels and CMAP amplitudes of the upper limbs: left median nerve (N=12, **A**), right median nerve (N=12, **B**), left ulnar nerve (N=10, **C**), right ulnar nerve (N=10, **D**). (**E**–**H**) Correlations between serum TIMP-1 levels and CMAP amplitudes of the lower limbs: left tibial nerve (N=12, **E**), right tibial nerve (N=12, **F**), left peroneal nerve (N=10, **G**), right peroneal nerve (N=10, **H**). Correlations were evaluated using Spearman's correlation coefficients. A *p*-value of <0.05 was considered significant. L: left; R: Right; Med: median; Per: peroneal.
